# The evaluation of cognitive-behavioral therapy-based intervention on type 2 diabetes patients with comorbid metabolic syndrome: a randomized controlled trial

**DOI:** 10.1186/s13098-023-01100-2

**Published:** 2023-07-17

**Authors:** Yanni Li, Nicholas Buys, Samantha Ferguson, Zhiyong Li, Yan-Chuan Shi, Li Li, Jing Sun

**Affiliations:** 1 Department of Endocrinology and Metabolism, Ningbo First Hospital, Ningbo, 315010 Zhejiang Province China; 2 School of Medicine and Dentistry, Griffith University, Gold Coast, QLD Q4222 Australia; 3 Griffith Health, Griffith University, Gold Coast, QLD Australia; 4 Institute for Integrated Intelligence and Systems, Griffith University, Gold Coast, QLD Australia; 5 School of Mechanical, Medical & Process Engineering, Queensland University of Technology, Brisbane, QLD Australia; 6 Neuroendocrinology Group, Garvan Institute of Medical Research, Faculty of Medicine and Health, University of New South Wales, 384 Victoria St, Darlinghurst, Australia; 7 Menzies Health Institute Queensland, Griffith University, Gold Coast, Australia

**Keywords:** Type 2 diabetes, Metabolic Syndrome, Cognitive Behavior Therapy, Health outcomes

## Abstract

**Background:**

Cognitive behavior therapy (CBT) has been applied in intervention research in diabetes patients with satisfying results. However, there was no research on type 2 diabetes (T2DM) patients with comorbidities. This study aimed to investigate the effectiveness of CBT on psychological variables, behavior variables, quality of life, sleep quality, and physical variables among adult T2DM patients with comorbid metabolic syndrome (MS).

**Methods:**

281 patients aged 18–75 years were recruited from Ningbo First Hospital in China from October 2021 to March 2022. Patients were randomized to the intervention group (IG, N = 148) or control group (CG, N = 133). Patients in the IG received 12 CBT-based sessions during a six-month intervention time. Patients in the CG received the usual care only. Univariate General Linear Model was used to analyze the effect of CBT-based interventions. The analysis was conducted by SPSS Version 28.

**Results:**

Results indicated that CBT-based intervention was superior in the following aspects: relieving depression symptoms: IG (4.11 ± 4.35 vs. 1.99 ± 2.12), CG (3.40 ± 3.26 vs. 2.32 ± 1.88), interaction effect (F = 4.074, P = 0.044); enhancing diabetes self-care behaviors: IG (26.79 ± 12.18 vs. 37.49 ± 10.83), CG (25.82 ± 13.71 vs. 31.96 ± 11.72), interaction effect (F = 5.242, P = 0.022); promoting the efficacy of CBT: IG (47.45 ± 6.83 vs. 50.76 ± 4.98), CG (46.74 ± 6.94 vs. 47.87 ± 5.11), interaction effect (F = 5.198, P = 0.023); improving subjective sleep quality: IG (0.93 ± 0.68 vs. 0.69 ± 0.63), CG (1.03 ± 0.72 vs. 1.01 ± 0.68), interaction effect (F = 3.927, P = 0.048).

**Conclusions:**

The CBT-based intervention was beneficial in improving depression symptoms, diabetes self-care behaviors, the efficacy of CBT, and sleep quality in T2DM patients with comorbid MS. The downtrend of body mass index, systolic blood pressure, diastolic pressure, and glycated hemoglobin was larger in the intervention group but not to a significant level.

*Trial registration*: This study has been prospectively registered at Australia New Zealand Clinical Trials Registry (Registration ID: ACTRN12621001348842 website: https://www.anzctr.org.au/trial/MyTrial.aspx).

**Supplementary Information:**

The online version contains supplementary material available at 10.1186/s13098-023-01100-2.

## Background

Diabetes mellitus (DM) is a chronic non-communicable disease characterized by hyperglycemia [1], caused by genetic, environmental, social, and lifestyle factors. Type 2 diabetes mellitus (T2DM) is the main type of DM, accounting for about 95% of individuals with DM [2]. The International Diabetes Federation (IDF) [3] estimated that there were about 451 million adult DM patients worldwide and the global health expenditure on DM patients was USD 850 billion in 2017, bringing a heavy economic burden to patients, their families, and society. Alarmingly, China has been the country with the largest number of DM patients [4], and the prevalence of DM is still rising [4]. Continuous hyperglycemia in DM patients is related to long-term functional impairment and chronic complications [5], threatening patients’ quality of life.

Metabolic syndrome (MS) refers to the pathological state in which the body's protein, fat, and carbohydrates are metabolically disordered [6]. The prevalence of MS differs across countries. Previous studies indicated that the prevalence of MS worldwide ranges from 26.7% to 30% [7–9], and there is no exception in the prevalence of MS in China. Based on data from China Nutrition and Health Surveillance (2015–2017), the standardized prevalence of the condition in China was 31.10% [10]. Patients with type 2 diabetes are more likely to have metabolic syndrome comorbidities including hypertension, dyslipidemia, or obesity, resulting in a large proportion of diabetes patients meeting the metabolic syndrome diagnostic criteria. MS is a risk factor for the development of diabetes and cardiovascular disease. The simultaneous presence of these two conditions could result in more cardiovascular disease and other adverse health consequences for patients. Type 2 diabetes and metabolic syndrome share a common pathogenesis in insulin resistance. Therefore, intervening in diabetes patients with comorbid MS is highly significant in terms of delaying the progression of diabetes and the onset of coronary heart disease.

Diabetes and metabolic syndrome are chronic and progressive diseases. The long duration of diabetes and metabolic syndrome increases the risk of developing psychological comorbidities, such as depression and anxiety [11]. Previous studies [12, 13] illustrated that the prevalence of depression and anxiety symptoms in DM was higher than in the general population. Concerning metabolic syndrome, previous research [14] revealed that metabolic syndrome patients had a higher risk of developing depression symptoms as well. This is worrying because comorbid depression or anxiety symptoms in DM and metabolic syndrome patients are related to nonadherence to medication[15], less self-care behaviors [16], increased risk of hyperglycemia [16], and reduced quality of life [17]. Therefore, effective prevention and alleviation of depression and anxiety symptoms in DM and metabolic syndrome patients are beneficial in delaying the further development of DM and the beginning of cardiovascular disease [18].

Additionally, good DM and metabolic syndrome management rely primarily on self-care on the part of patients[19]. Previous studies illustrated that self-management programs were beneficial in improving patients’ self-care behaviors [20, 21], including dietary behaviors [22], physical activity [23], and medication adherence [24]. However, the majority of diabetes and metabolic syndrome patients have underlying psychological distress and anxiety that may prevent them from improving behaviors related to these lifestyles. Therefore, ways to alleviate the negative emotions in diabetes and metabolic syndrome patients are significant in improving self-care behaviors.

Cognitive behavior therapy (CBT) is a short-term and structured therapy. It aims to change an individual’s problematic cognitions by changing thinking or beliefs and behaviors and to make these less destructive [25]. CBT aims to improve patients' abilities to cope with maladaptive cognitions and/or behavioral patterns [26, 27]. Numerous studies [27–29] have investigated the efficacy of CBT on DM and metabolic syndrome patients and identified satisfying results in improving health outcomes. Jenkinson and colleagues [30] found that CBT therapy reduced depression and distress symptoms in diabetes patients. Likewise, Newby and colleagues [27] concluded that there were significant differences in depression symptoms and mental well-being of quality of life between the web-based CBT group and the control group. Similarly, Uchendu and Blake [28] proposed that CBT helped improve glycaemic control and depression symptoms in DM patients. When it comes to the effectiveness of CBT in patients with MS, Garcia-Silva and colleagues [31] found that CBT was beneficial in reducing waist circumference, triglycerides, and adherence to the Mediterranean Diet in MS patients when compared to the control group. Zhang and colleagues [32] conducted a randomized controlled trial in cardio-metabolic syndrome patients and results demonstrated that CBT-based intervention could improve the physical and mental health conditions among this group of patients.

The number of DM patients in China is increasing rapidly, and the proportion of DM patients meeting the diagnostic criteria for metabolic syndrome (MS) is large [33]. DM patients with comorbid MS may have an increased risk of cardiovascular events [33]. However, no previous studies were conducted on T2DM patients with comorbid MS. This study aimed to conduct a randomized controlled trial in Ningbo First Hospital, China, to comprehensively evaluate the effectiveness of CBT-based intervention on health outcomes in T2DM patients with comorbid MS. The research hypothesis was that CBT-based intervention was beneficial in improving psychological and behavioral variables, including depression symptoms, anxiety symptoms, diabetes knowledge, the efficacy of CBT, diabetes self-care behaviors; quality of life, and sleep quality; physiological variables, including body mass index (kg/m^2^), systolic blood pressure (mmHg), diastolic blood pressure (mmHg), glycated hemoglobin (%), fasting plasma glucose (mmol/L), triglycerides (mmol/L), total cholesterol (mmol/L), high-density lipoprotein cholesterol (mmol/L), and low-density lipoprotein cholesterol (mmol/L).

## Methods

### Study population

#### Inclusion and exclusion criteria

Endocrine outpatients or inpatients were recruited if they met the following criteria: aged 18–75 years old, diagnosed with T2DM with comorbid MS, did not participate in similar intervention programs, signed an informed consent form, and were willing to participate in this study. Patients were excluded if they had type 1 diabetes, gestational diabetes, or any other special type of diabetes, had advanced diabetes complications, had a severe mental illness, or could not use mobile phones.

The diagnosis criteria for diabetes referred to the report of the WHO Diabetes Expert Committee [34] which was as follows: typical symptoms of diabetes plus either (1) random blood glucose ≥ 11.1 mmol/L; (2) fasting blood glucose ≥ 7.0 mmol/L; (3) oral glucose tolerance test 2 h ≥ 11.1 mmol/L. The diagnosis criteria for MS referred to the IDF standard [35] including abdominal obesity, waist circumference ≥ 90 cm (Chinese male) or ≥ 80 cm (Chinese female), and those with two or more of the following characteristics: 1) triglycerides > 1.70 mmol/L or have received relevant treatment; 2) high-density lipoprotein cholesterol < 1.03 mmol/L (male) or < 1.29 mmol/L (female), or have received relevant treatment; 3) systolic blood pressure ≥ 130 or diastolic blood pressure ≥ 85 mmHg, or have received relevant treatment; 4) fasting blood glucose ≥ 5.6 mmol/L or diagnosed with T2DM previously.

#### Sample size

The glycated hemoglobin value was chosen to calculate the sample size. The ratio of intervention and control group sample was set as 1:1. The formula to estimate the sample size for the study was as follows [36]:

$$N=\frac{2{S}^{2}(Z\alpha \text{/2}+Z\beta {)}^{2}}{{\upsigma }^{2}}$$

N represented the sample size per group; Z_α/2_ and Z_β_ represented the standard normal deviates for type I and type II errors; S represented the squared standard deviation, and σ^2^ represented the squared difference between the treatment and control groups. By referring to the previous paper [37, 38], S was set as 0.700%, σ as 0.275%, α as 0.05, β as 0.80. The sample size for each group was calculated as 102. Considering the nonresponse (20%) and attrition (15%) conditions, the sample size was calculated as 140 in the intervention group and 140 in the control group, bringing the total number of patients to 280.

#### Randomized grouping

Random integers were generated by the SPSS 28 software. Visual Binning was used to randomize the random integers into the intervention group or the control group based on the ratio of 1:1. Patients were randomly assigned to either the intervention group or control group according to the enrollment time. Ten to fifteen patients formed one group of the intervention or control group. A social media group was established accordingly. Due to the nature of the study, the therapists and patients were not blinded. But the data analysts did not know the grouping results and were blinded.

#### Intervention methods

Patients in the intervention group received the CBT-based intervention and the usual care. The CBT-based intervention included 12 manual-based sessions during the six-month intervention time. Each CBT intervention lasted for 20–30 min. There were eight weekly sessions in the first two months and followed by four monthly sessions in the following months. Considering that some patients lived far away from the hospital and could not go to the endocrinology clinic to participate in all the sessions, this study adopted the combination of face-to-face and online intervention modes. The fourth session (at the end of the first month), the ninth session (at the end of the third month), and the twelfth session (at the end of the sixth month) were held in the endocrinology clinic face-to-face, which was arranged with patients’ regular visits to the hospital. The rest of the sessions were conducted through social media groups online. The one-on-one intervention was carried out in person and followed the guidelines provided in the intervention manual (Additional file 1: Figure S1). The social media group hosted the online session where the intervention content was shared and discussions were facilitated through questions and answers within the group. A quiz was shared with the social media group following the session to enhance comprehension of the intervention material and monitor the patients' adherence to the online session.

Patients belonging to the intervention group were deemed to have participated in the online intervention session if they completed the quiz within three days or engaged in group discussions. In addition, they were categorized as having participated in the face-to-face intervention session if they visited the hospital for their regular review and partook in the 20–30-min intervention sessions within two weeks, adhering to the intervention protocol. Among the intervention group, 127 patients participated in at least eight intervention sessions and were considered as having completed the intervention project. The participation detail for intervention group patients was presented in Additional file 1: Table S1.

The usual care referred to the health education at baseline in the endocrinology clinic, including suggestions on healthy eating and scientific exercise according to the Guidelines for the Prevention and Treatment of Type 2 Diabetes in China (2020 Edition). For patients in the control group, there was only one 20–30 min face-to-face usual care session at the clinic at the baseline time, conducted by the therapist. There were no further intervention sessions conducted for the duration of the six months (Additional file 1: Figure S1). All patients in the control group attended the single usual care session at baseline. In addition, patients visited doctors every three months for patients in both the intervention and control groups. For each usual care visit, it was approximately 20 min.

Yanni Li served as the therapist, with the additional involvement of endocrinologist Ye Zhou to provide expertise on endocrinology-related consultations to patients. Both Yanni Li and Ye Zhou were trained before the research and the intervention was conducted in strict accordance with the intervention protocol. The intervention session contents were in Additional file 1: Table S2.

#### Measurement

The measurement of this study included psychological and behavioral variables: depression symptoms, anxiety symptoms, diabetes knowledge, the efficacy of CBT, and diabetes self-care behaviors; quality of life and sleep quality; physiological variables: body mass index (BMI, kg/m^2^), systolic blood pressure (SBP, mmHg), diastolic blood pressure (DBP, mmHg), glycated hemoglobin (HbA_1c_, %), fasting plasma glucose (FPG, mmol/L), triglycerides (TG, mmol/L), total cholesterol (TC, mmol/L), high-density lipoprotein cholesterol (HDL-C, mmol/L), and low-density lipoprotein cholesterol (LDL-C, mmol/L). The primary outcomes were HbA_1c_, depression and anxiety symptoms, and diabetes self-care behaviors. The secondary outcomes were diabetes knowledge, the efficacy of CBT, quality of life, sleep quality, BMI, blood pressure, and blood lipids.

#### Depression symptoms

Patients’ depression symptoms were assessed by the Patient Health Questionnaire-9 (PHQ-9) [39]. It was a self-report tool that evaluate patients’ depression symptoms during the previous two weeks. There were nine items, and each item had a score of zero to three. The total score ranged from zero to 27. A higher score indicated a worse depression condition. The Cronbach’s Alpha level was 0.764 for this study.

#### Anxiety symptoms

Patients’ anxiety symptoms were assessed by the General Anxiety Disorder-7 (GAD-7) [40]. It was a self-report tool that evaluate patients’ anxiety symptoms during the previous two weeks. There were seven items, and each item had a score of zero to three. The total score ranged from zero to 21. A higher score represented a worse anxiety condition. The Cronbach’s Alpha level was 0.887 for this study.

#### Diabetes self-care

Patients’ diabetes self-care ability was evaluated by the Summary of Diabetes Self-Care Activities Questionnaire (SDSCA) [41]. It was a self-report tool to assess patients’ diabetes self-care during the previous week. There were five dimensions in terms of diabetes diet, physical activity, self-monitoring of blood glucose, foot care, and smoking. Patients were asked, “In the past seven days, how many days did you engage in each of the above activities?”. The number of days was recorded as the score of this item, and the total score ranged from zero to 70. A higher score indicated better self-care. The Cronbach’s Alpha level was 0.666 for this study.

#### Diabetes knowledge

Patients’ diabetes knowledge was assessed by The Diabetes Knowledge Scale [42]. It had 10 items, and topics included the scientific fasting blood glucose value; common symptoms of DM; DM complications; self-monitoring of blood glucose; causes of hypoglycemia; principles of a healthy diet for DM patients; principles of a healthy exercise plan for DM patients. Patients got one point if they chose the right answer and the total score ranged from zero to 10. The Cronbach’s Alpha level was 0.716 for this study.

#### The efficacy of CBT

Patients’ efficacy of CBT was evaluated by the self-designed questionnaire named “Evaluation for the Effectiveness of Cognitive Behavioral Therapy (EECBT)” based on a previous study [43]. There were 12 self-rated items, and each item had a score of zero to five. The total score ranged from zero to 60. A higher score indicated the higher efficacy of CBT. There were three items related to the thinking characteristics in dealing with diabetes; five items related to the behavior characteristics in dealing with diabetes; two items related to the ability to regulate emotions in daily life; two items reflected the ability to cope with difficulties in daily life. The Cronbach’s Alpha level was 0.764 for this study.

#### Quality of life

Patients’ quality of life during the past month was evaluated by the SF-12 Quality of Life Questionnaire [44]. It had two domains of physical health and mental health. All scores were converted into standard scores, which ranged from zero to 100. A higher score indicated a better quality of life. The Cronbach’s Alpha level was 0.766 for this study.

#### Sleep quality

Patients’ sleep quality was assessed by the Pittsburgh Sleep Quality Index (PSQI) [45]. It was a self-report questionnaire evaluating patients’ sleep conditions during the previous month. There were 19 items and seven subscales of subjective sleep quality, sleep latency, sleep duration, habitual sleep efficiency, sleep disturbances, use of sleep medications, and daytime dysfunction. Each subscale scored between zero to three, and the sum of seven subscales scores gave the PSQI total score. A higher score indicated a poorer sleep quality.

#### Satisfaction with the intervention research

Patients’ satisfaction with the intervention program was evaluated by the self-designed questionnaire. It was a self-reported questionnaire with 10 items. A higher score indicated higher satisfaction with this research. There was one item related to overall satisfaction; three items about skills and attitudes of therapists; two items on the intervention contents; two items on the intervention schedule and mode; two items about self-improvement. The Cronbach’s Alpha level was 0.986 for this study.

#### Physiological health outcomes

Physiological variables, including glycated hemoglobin HbA_1c_ (%), fasting blood glucose FBP (mmol/L), triglycerides TG (mmol/L), total cholesterol TC (mmol/L), high-density lipoprotein cholesterol HDL-C (mmol/L), and low-density lipoprotein cholesterol LDL-C (mmol/L) were obtained from patients’ medical records. Systolic blood pressure SBP (mmHg), diastolic blood pressure DBP (mmHg), height (cm), and weight (kg) were measured at the clinic by the researcher. The lifestyle-related variables of smoking cigarettes and drinking alcohol, and the disease-related variables of treatment methods, years of DM, and DM implications were obtained by a self-designed questionnaire.

#### Data collection

The data was collected at the baseline and the end of the intervention (6th month).

The depression and anxiety symptoms, diabetes knowledge, the efficacy of cognitive behavior therapy, diabetes self-care behaviors, quality of life, and sleep quality were obtained through an online survey at baseline and 6 months The weight, height, systolic blood pressure, and diastolic blood pressure of patients were measured at baseline and 6 months by the researcher at the clinic. The values of HbA_1c_, fasting blood glucose, triglycerides, total cholesterol, low-density lipoprotein, and high-density lipoprotein, were obtained from the patient’s medical records at baseline and 6 months.

#### Statistical analysis

Descriptive analyses were presented as the mean and standard deviation for continuous variables. Independent Two Sample T Test was used to evaluate the different characteristics of patients in the intervention group and control group in terms of continuous variables. In addition, descriptive analyses were presented as numbers and percentages for categorical variables. The Chi-square Test was used to assess the different characteristics of patients in the two groups in terms of categorical variables.

The potential confounding variables were age, sex, marriage, education level, monthly income, smoking, drinking, treatment method, years of diabetes, and diabetes complications. The continuous variables with significant differences between the intervention group and control group by the Independent Two Sample T Test were included as covariates. The categorical variables with significant differences between the intervention group and control group by the Chi-square Test were included as the fixed factors. Mean Imputation was used to replace the missing data. Univariate General Linear Model was used to analyze the effect of CBT-based interventions, including the effect of time, the effect of the group, and the interaction effect of time and group. The dependent variables were psychological and behavioral variables, including depression and anxiety symptoms (score), diabetes knowledge (score), the efficacy of CBT (score), diabetes self-care abilities (score), quality of life (score), and sleep quality (score); physiological variables were body mass index (BMI kg/m^2^), SBP (mmHg), DBP (mmHg), HbA_1c_ (%), FBG (mmol/L), TG (mmol/L), TC (mmol/L), HDL-C (mmol/L), and LDL-C (mmol/L). The fixed factors were the time (pre-intervention time vs. post-intervention time) and different intervention methods (CBT-based intervention group vs. usual care group).

The IBM SPSS Statistics Version 28 was used for all analyses. The two-sided test and P < 0.05 were considered statistically significant.

## Results

### Basic patients’ characteristics

A total of 281 patients were included in this study, with 148 in the intervention group and 133 in the control group. The overall dropout rate was 14.23%. There were significant differences in demographic characteristics of age, education level, and diabetes complications between the intervention group and control group. Patients in the intervention group were significantly younger than those in the control group (47.66 ± 12.31 vs. 52.41 ± 10.80, T = -3.423, P = 0.001). The proportion of patients with a college and above education level was larger in the intervention group (61.60% vs. 38.40%, χ^2^ = 7.517, P = 0.023). More patients in the intervention group did not have a diabetes complication when compared to the control group (56.70% vs. 43.30%, χ^2^ = 6.098, P = 0.014). In addition, there were also significant differences between the two groups in diabetes knowledge and sleep quality. Patients in the intervention group scored higher diabetes knowledge scores than those in the control group (5.07 ± 2.19 vs. 4.32 ± 2.26, T = 2.829, P = 0.005). Intervention group patients had lower PSQI scores (indicating better sleep quality) than those in the control group (6.22 ± 3.21 vs. 7.08 ± 3.31, T = -2.228, P = 0.027). Therefore, the confounding factors were age, education level, and diabetes complications. The continuous variable (age) was included as the covariate and the categorical variables (education level and diabetes complications) were included as the fixed factors in the General Linear Model. The differences between other variables were not statistically significant **(**Table 1**).**

**Table 1:** Distribution of patients’ general characteristics in the intervention group and control group

Variables	Total	Intervention (N = 148)	Control (N = 133)	T/χ^2^	P
Age	49.91 ± 11.84	47.66 ± 12.31	52.41 ± 10.80	− 3.423	0.001
Gender				0.734	0.392
Female	113(40.20)	56(49.60)	57(50.40)		
Male	168(59.80)	92(54.80)	76(45.20)		
Marriage				5.289	0.071
Unmarried	16(5.70)	12(75.00)	4(25.00)		
Married with a spouse	255(90.70)	133(52.20)	122(47.80)		
Divorced	10(3.60)	3(30.00)	7(70.00)		
Education				7.517	0.023
Junior high school and below	116(41.30)	50(43.10)	66(56.90)		
High school or junior college	92(32.70)	53(57.60)	39(42.40)		
College and above	73(26.00)	45(61.60)	28(38.40)		
Monthly income (RMB)				2.352	0.308
< 5000	97(34.50)	47(48.50)	50(51.50)		
5000–10,000	95(33.80)	56(58.90)	39(41.10)		
> 10,000	89(31.70)	45(50.60)	44(49.40)		
Smoke				0.860	0.354
No	212(75.40)	115(54.20)	97(45.80)		
Yes	69(24.60)	33(47.80)	36(52.20)		
Drink				0.603	0.438
No	184(65.50)	100(54.30)	84(45.70)		
Yes	97(34.50)	48(49.50)	49(50.50)		
Treatment				2.104	0.349
Oral medicine	90(32.00)	53(58.90)	37(41.10)		
Oral medicine and insulin	35(12.50)	18(51.40)	17(48.60)		
Oral medicine and GLP1	156(55.50)	77(49.40)	79(50.60)		
Years of diabetes				3.603	0.058
≤ 5	189(67.30)	107(56.60)	82(43.40)		
> 5	92(32.70)	41(44.60)	51(55.40)		
Complications				6.098	0.014
No	215(76.50)	122(56.70)	93(43.30)		
Yes	66(23.50)	26(39.40)	40(60.60)		
Depression (Score)	3.78 ± 3.88	4.11 ± 4.35	3.40 ± 3.26	1.548	0.123
Anxiety (Score)	2.46 ± 3.51	2.76 ± 3.86	2.13 ± 3.04	1.540	0.125
SDSCA (Score)	26.33 ± 12.91	26.79 ± 12.18	25.82 ± 13.71	0.629	0.530
SF12 (Score)	72.24 ± 13.37	72.49 ± 12.93	71.95 ± 13.88	0.341	0.733
PSQI (Score)	6.63 ± 3.28	6.22 ± 3.21	7.08 ± 3.31	− 2.228	0.027
Diabetes knowledge (Score)	4.72 ± 2.25	5.07 ± 2.19	4.32 ± 2.26	2.829	0.005
Efficacy of CBT (Score)	47.11 ± 6.88	47.45 ± 6.83	46.74 ± 6.94	0.862	0.389
BMI (kg/m^2^)	27.13 ± 3.14	27.16 ± 3.43	27.10 ± 2.81	0.147	0.883
SBP (mmHg)	137.54 ± 16.79	138.04 ± 17.37	136.98 ± 16.17	0.523	0.602
DBP (mmHg)	82.04 ± 10.66	82.88 ± 11.48	81.10 ± 9.63	1.414	0.158
HbA_1c_ (%)	7.38 ± 1.70	7.36 ± 1.59	7.40 ± 1.82	− 0.172	0.863
FPG (mmol/L)	8.20 ± 3.30	8.16 ± 2.62	8.25 ± 3.94	− 0.248	0.804
TG (mmol/L)	2.07 ± 1.80	2.06 ± 1.84	2.07 ± 1.75	− 0.050	0.960
TC (mmol/L)	4.90 ± 1.33	4.85 ± 1.30	4.96 ± 1.37	− 0.665	0.507
HDL-C (mmol/L)	1.18 ± 0.24	1.14 ± 0.22	1.21 ± 0.27	− 2.418	0.016
LDL-C (mmol/L)	3.08 ± 0.96	3.08 ± 0.92	3.08 ± 1.00	− 0.002	0.998

*RMB* Ren min bi, *GLP-1RA* Glp-1 receptor agonist, *SDSCA* Summary of Diabetes Self-Care Activities, *CBT* Cognitive behavioral therapy, *SF12* Short Form 12, *PSQI* Pittsburgh Sleep Quality Index, *BMI* Body mass index, *SBP* Systolic blood pressure, *DBP* Diastolic blood pressure, *HbA*_*1c*_ Glycated hemoglobin, *FPG* Fasting plasma glucose, *TG* Triglycerides, *TC* Total cholesterol, *HDL-C* High-density lipoprotein cholesterol, *LDL-C* Low-density lipoprotein cholesterol

### Effects of CBT-based intervention on psychological variables, diabetes knowledge, and efficacy of CBT

**Fig. 1:**
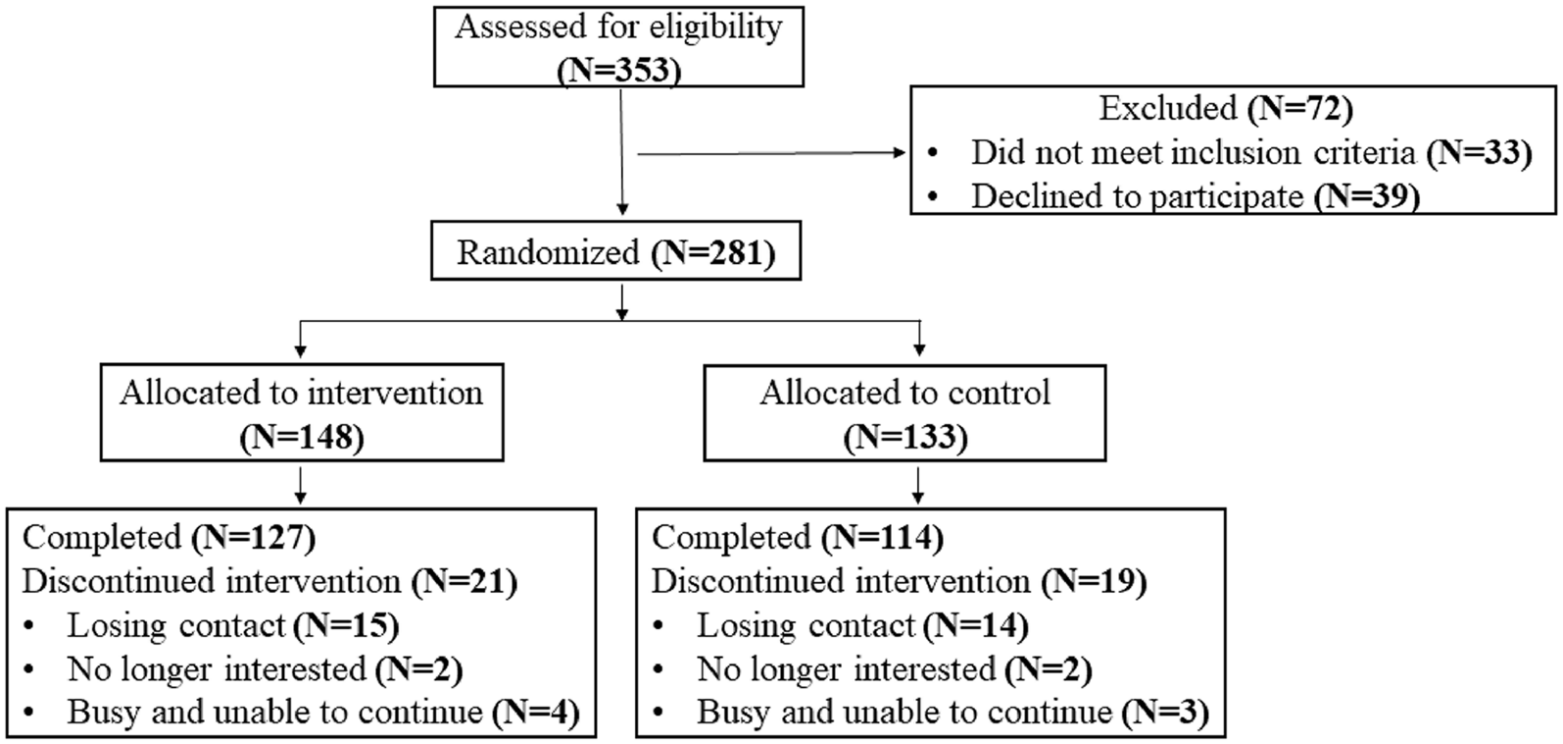
CONSORT Diagram

The interaction effect for depression symptoms was statistically significant (F = 4.074, P = 0.044), indicating that the intervention group exhibited considerably greater improvement in depression symptoms (Fig. 1) as compared to the control group. There was also a significant time effect for depression symptoms (F = 38.291, P < 0.001) and depression scores decreased significantly both in the intervention group (4.11 ± 4.35 vs. 1.99 ± 2.12) and the control group (3.40 ± 3.26 vs. 2.32 ± 1.88) after the intervention time. In addition, the interaction effect for anxiety symptoms was not significant (F = 0.731, P = 0.393). However, there was a significant time effect for anxiety symptoms (F = 23.057, P < 0.001), and the anxiety scores reduced significantly both in the intervention group (2.76 ± 3.86 vs. 1.41 ± 2.13) and control group (2.13 ± 3.04 vs. 1.18 ± 1.97) after the intervention time (Table 2).

**Table 2:** Differences between pre-intervention and post-intervention time in the intervention group and control group of physiological and psychological indicators

Variables	N		Pre-intervention		Post-intervention		F (P value)			
			M(SD)		M(SD)		Time		Group	Interaction
Depression (Score)						38.291(< 0.001)		0.344(0.558)		4.074(0.044)
Intervention	148		4.11 ± 4.35		1.99 ± 2.12					
Control	133		3.40 ± 3.26		2.32 ± 1.88					
Anxiety (Score)						23.057(< 0.001)		1.863(0.173)		0.731(0.393)
Intervention	148		2.76 ± 3.86		1.41 ± 2.13					
Control	133		2.13 ± 3.04		1.18 ± 1.97					
SDSCA (Score)						71.281(< 0.001)		17.099(< 0.001)		5.242(0.022)
Intervention	148		26.79 ± 12.18		37.49 ± 10.83					
Control	133		25.82 ± 13.71		31.96 ± 11.72					
General diet (Score)						334.949(< 0.001)		22.988(< 0.001)		7.691(0.006)
Intervention	148		3.38 ± 2.54		10.13 ± 4.27					
Control	133		2.79 ± 2.93		7.77 ± 5.04					
Special diet (Score)						33.528(< 0.001)		0.525(0.469)		1.349(0.246)
Intervention	148		7.95 ± 3.97		10.09 ± 3.54					
Control	133		8.17 ± 3.89		9.59 ± 3.22					
Whether to exercise (Score)						153.332(< 0.001)		5.189(0.023)		1.340(0.248)
Intervention	148		3.53 ± 2.55		8.43 ± 5.32					
Control	133		3.44 ± 2.98		7.50 ± 5.77					
Self-monitoring of BG (Score)						29.843(< 0.001)		4.371(0.037)		3.443(0.064)
Intervention	148		1.70 ± 2.19		3.56 ± 3.79					
Control	133		1.77 ± 2.46		2.69 ± 3.38					
Foot care (Score)							217.478(< 0.001)		5.459(0.020)	4.127(0.043)
Intervention	148		0.82 ± 1.54		4.73 ± 3.76					
Control	133		0.83 ± 1.53		3.79 ± 3.38					
Diabetes knowledge (Score)						49.779(< 0.001)		2.724(0.099)		2.469(0.117)
Intervention	148		5.07 ± 2.19		6.04 ± 2.05					
Control	133		4.32 ± 2.26		5.84 ± 2.04					
Efficacy of CBT (Score)						21.755(< 0.001)		5.003(0.026)		5.198(0.023)
Intervention	148		47.45 ± 6.83		50.76 ± 4.98					
Control	133		46.74 ± 6.94		47.87 ± 5.11					
SF-12 (Score)							5.042(0.025)		2.048(0.153)	1.894(0.169)
Intervention	148		72.49 ± 12.93		76.06 ± 8.87					
Control	133		71.95 ± 13.88		72.80 ± 10.49					
PCS (Score)							0.005(0.943)		1.473(0.225)	0.179(0.672)
Intervention	148		68.80 ± 13.27		69.36 ± 11.14					
Control	133		66.95 ± 16.21		66.56 ± 13.42					
MCS (Score)							13.981(< 0.001)		1.540(0.215)	3.682(0.056)
Intervention	148		76.18 ± 16.95		82.76 ± 10.29					
Control	133		76.94 ± 16.07		79.05 ± 10.36					
PSQI (Score)							5.245(0.022)		7.020(0.008)	1.179(0.278)
Intervention	148		6.22 ± 3.21		5.34 ± 3.02					
Control	133		7.08 ± 3.31		6.77 ± 3.32					
A-Sleep quality (Score)						5.385(0.021)		6.075(0.014)		3.927(0.048)
Intervention	148		0.93 ± 0.68		0.69 ± 0.63					
Control	133		1.03 ± 0.72		1.01 ± 0.68					
B-Time to fall asleep (Score)						0.087(0.768)		1.335(0.248)		0.001(0.980)
Intervention	148		0.80 ± 1.11		0.77 ± 0.90					
Control	133		0.99 ± 1.16		0.97 ± 1.00					
C-Sleep time (Score)						1.203(0.273)		7.344(0.007)		0.062(0.803)
Intervention	148		0.89 ± 0.70		0.93 ± 0.62					
Control	133		1.07 ± 0.69		1.14 ± 0.68					
D-Sleep efficiency (Score)						9.409(0.002)		1.727(0.189)		0.356(0.551)
Intervention	148		0.93 ± 1.03		0.73 ± 0.87					
Control	133		1.21 ± 1.16		0.91 ± 0.96					
E-Sleep disorder (Score)						7.707(0.006)		2.029(0.155)		0.679(0.410)
Intervention	148		1.11 ± 0.52		1.04 ± 0.44					
Control	133		1.25 ± 0.47		1.11 ± 0.38					
F-Hypnotic drugs (Score)						0.841(0.359)		0.013(0.908)		0.669(0.414)
Intervention	148		0.07 ± 0.40		0.07 ± 0.37					
Control	133		0.08 ± 0.41		0.15 ± 0.59					
G-Day time dysfunction (Score)						5.620(0.018)		3.088(0.079)		6.793(0.009)
Intervention	148		1.49 ± 0.93		1.10 ± 0.82					
Control	133		1.46 ± 0.93		1.48 ± 0.96					
BMI (kg/m^2^)							25.639(< 0.001)		2.890(0.090)	0.208(0.649)
Intervention	148		27.16 ± 3.43		25.80 ± 3.09					
Control	133		27.10 ± 2.81		25.97 ± 2.68					
SBP (mmHg)							63.549(< 0.001)		0.026(0.872)	1.694(0.194)
Intervention	148		138.04 ± 17.37		125.80 ± 13.41					
Control	133		136.98 ± 16.17		128.19 ± 15.49					
DBP (mmHg)							45.485(< 0.001)		0.085(0.771)	2.041(0.154)
Intervention	148		82.88 ± 11.48		76.10 ± 8.37					
Control	133		81.10 ± 9.63		76.69 ± 10.47					
HbA_1C_ (%)							41.934(< 0.001)		0.065(0.799)	0.090(0.764)
Intervention	148		7.36 ± 1.59		6.51 ± 1.27					
Control	133		7.40 ± 1.82		6.62 ± 1.23					
FBG (mmol/L)							46.569(< 0.001)		0.001(0.982)	0.097(0.755)
Intervention	148		8.16 ± 2.62		6.65 ± 2.21					
Control	133		8.25 ± 3.94		6.61 ± 1.83					
TG (mmol/L)							3.916(0.048)		1.121(0.290)	0.025(0.876)
Intervention	148		2.06 ± 1.84		1.77 ± 2.04					
Control	133		2.07 ± 1.75		1.74 ± 2.01					
TC (mmol/L)							39.899(< 0.001)		5.850(0.016)	0.055(0.815)
Intervention	148		4.85 ± 1.30		4.25 ± 1.11					
Control	133		4.96 ± 1.37		4.31 ± 1.09					
HDL_C (mmol/L)							2.016(0.156)		3.710(0.055)	0.804(0.370)
Intervention	148		1.14 ± 0.22		1.16 ± 0.19					
Control	133		1.21 ± 0.27		1.30 ± 0.89					
LDL_C (mmol/L)							53.314(< 0.001)		2.998(0.084)	0.004(0.952)
Intervention	148		3.08 ± 0.92		2.58 ± 0.65					
Control	133		3.08 ± 1.00		2.59 ± 0.78					

*SDSCA* Summary of Diabetes Self-Care Activities, *BG* blood glucose, *CBT* Cognitive behavioral therapy, *SF12* Short Form 12, *PCS* Physical Component Summary, *MCS* Mental Component Summary, *PSQI* Pittsburgh Sleep Quality Index, *BMI* Body mass index, *SBP* Systolic blood pressure, *DBP* Diastolic blood pressure, *HbA*_*1c*_ Glycated hemoglobin, *FPG* Fasting plasma glucose, *TG* Triglycerides, *TC* Total cholesterol, *HDL-C* High-density lipoprotein cholesterol, *LDL-C* Low-density lipoprotein cholesterol

The interaction effect for diabetes knowledge was not significant (F = 2.469, P = 0.117). However, there was a significant time effect (F = 49.779, P < 0.001) and there was an increase in diabetes knowledge scores in the intervention group (5.07 ± 2.19 vs. 6.04 ± 2.05) and control group (4.32 ± 2.26 vs. 5.84 ± 2.04) after the intervention time. In addition, the interaction effect for the efficacy of CBT was significant (F = 5.198, P = 0.023), indicating the intervention group (47.45 ± 6.83 vs. 50.76 ± 4.98) improved more in the efficacy of CBT when compared to the control group (46.74 ± 6.94 vs. 47.87 ± 5.11). There was also a significant time effect on the efficacy of CBT and both groups scored higher in the efficacy of CBT after the intervention time (F = 21.755, P < 0.001) **(**Table 2**).**

### Effects of CBT-based intervention on behavioral variables

When it came to the diabetes self-care behavior variables, there was a significant interaction effect (F = 5.242, P = 0.022) for the total SDSCA scores, indicating that overall diabetes self-care behaviors improved more in the intervention group when compared to the control group. There was also a significant time effect (F = 71.281, P < 0.001), and the SDSCA scores increased significantly in both the intervention group (26.79 ± 12.18 vs. 37.49 ± 10.83) and control group (25.82 ± 13.71 vs. 31.96 ± 11.72) after the intervention time. Additionally, there was a significant interaction effect for the general diet subscale (F = 7.691, P = 0.006), predicting that intervention group patients improved more significantly in the general diet. There was also a significant time effect (F = 334.949, P < 0.001) and general diet scores increased significantly both in the intervention group (3.38 ± 2.54 vs. 10.13 ± 4.27) and control group (2.79 ± 2.93 vs. 7.77 ± 5.04) after the intervention time. Likewise, there was also a significant interaction effect for the foot care (F = 4.127, P = 0.043), illustrating intervention patients improved more significantly in the foot care behaviors **(**Table 2**).**

### Effects of CBT-based intervention on quality of life and quality of sleep

As for the quality of life, there was not a significant interaction effect for the quality of life (F = 1.894, P = 0.169). However, the time effect for the quality of life was significant (F = 5.042, P = 0.025) and patients in the intervention group (72.49 ± 12.93 vs. 76.06 ± 8.87) and the control group (71.95 ± 13.88 vs. 72.80 ± 10.49) scored higher after the intervention time. In addition, there was a significant time effect on the mental well-being of quality of life (F = 13.981, P < 0.001), demonstrating both group patients scored higher after the intervention time **(**Table 2**).**

When it came to sleep quality, there was not a significant interaction effect for the overall PSQI scores (F = 1.179, P = 0.278). However, the time effect was significant (F = 5.245, P = 0.022) and PSQI scores decreased significantly in the intervention group (6.22 ± 3.21 vs. 5.34 ± 3.02) and control group (7.08 ± 3.31 vs.6.77 ± 3.32) after the intervention time. In addition, there was a significant interaction effect for the subjective sleep quality (F = 3.927, P = 0.048) and daytime dysfunction (F = 6.793, P = 0.009), indicating the intervention group patients improved more significantly in these two subscales of PSQI. **(**Table 2**).**

### Effects of CBT-based intervention on physiological variables

There was not a significant interaction effect for BMI (F = 0.208, P = 0.649). But there was a significant time effect (F = 25.639, P < 0.001), and BMI decreased significantly in both the intervention group (27.16 ± 3.43 vs. 25.80 ± 3.09) and control group (27.10 ± 2.81 vs. 25.97 ± 2.68) after the intervention time. The downward trend in the intervention group was greater than in the control group. Similarly, there was not a significant interaction effect for SBP (F = 1.694, P = 0.194). However, the significant time effect (F = 63.549, P < 0.001) indicated that intervention group patients (138.04 ± 17.37 vs. 125.80 ± 13.41) had a larger downward trend in SBP than those in the control group (136.98 ± 16.17 vs. 128.19 ± 15.49). Likewise, the significant time effect for DBP (F = 45.485, P < 0.001) indicated that intervention group patients had a larger downward trend in DBP. As for the HbA_1c_, there was not a significant interaction effect (F = 0.090, P = 0.764). But the time effect was significant (F = 41.934, P < 0.001), and patients in the intervention group (7.36 ± 1.59 vs. 6.51 ± 1.27) and control group (7.40 ± 1.82 vs. 6.62 ± 1.23) had significant decreased HbA_1c_ values after the intervention time. Patients in the intervention group decreased more but not to a significant level. When it came to TG, TC, and LDL-C, there were no significant interaction effects for TG (F = 0.025, P = 0.876), TC (F = 0.055 P = 0.815), or LDL-C (F = 0.004, P = 0.952). But the time effects for TG (F = 3.916, P = 0.048), TC (F = 39.899, P < 0.001), and LDL-C (F = 53.314, P < 0.001) were significant.

## Discussion

This study assessed the efficacy of CBT-based interventions on T2DM patients with comorbid MS. The findings can be summarized as follows: CBT interventions were more effective in reducing depression symptoms and improving diabetes self-management behaviors (including diet and foot care), as well as enhancing sleep quality. However, changes in BMI, HbA_1c_, SBP, and DBP in the intervention group were not statistically significant.

### Effects of CBT-based intervention on psychological and behaviors variables

This study demonstrated that CBT-based intervention was beneficial in relieving depression symptoms, and diabetes self-care behaviors in T2DM patients with comorbid MS when compared to the usual care control group. Likewise, there were similar research results in previous studies. Uchendu and Blake [28] outlined that CBT was beneficial in easing depression in diabetes adults in a systematic review and meta-analysis. Andreae and colleagues [46] argued that CBT-based programs improved depressive symptoms in individuals with diabetes and chronic pain in a randomized controlled trial. In addition, Jaqueline and colleagues [47] investigated the effectiveness of CBT on patients with MS and results indicated that intervention group patients improved more in negative emotions of anger. Previous research [48] has shown that individuals with type 2 diabetes and metabolic syndrome who experience psychological stress are at risk of exhibiting inadequate self-care behaviors, which can lead to poor management of their condition. The positive effects of CBT on improving negative emotions in our study could be attributed to the effects of CBT in identifying and replacing dysfunctional cognitions with positive and functional thoughts [49]. In other words, CBT strategies used in intervention sessions, including the cognitive triangle, cognitive restructuring, and behavioral activation, enabled participants to recognize their automatic thinking and cognitive distortions. Gradually, participants could learn to improve their dysfunctional cognitions [49]. Therefore, CBT-based intervention is a therapy method for mitigating depression symptoms in T2DM patients with comorbid MS in conjunction with medication-based treatment, and it could be applied in diabetes management to enhance participants’ mental health.

When it came to diabetes self-care behaviors, Clarke and colleagues [50] proved that T2DM patients in the CBT intervention group showed significant improvements in blood glucose monitoring and medication adherence. Safren and colleagues [51] illustrated that diabetes patients in the CBT intervention group had better adherence to glucose monitoring than those in the treatment-as-usual group. Additionally, CBT intervention was shown by Jaqueline and her colleagues [31] to enhance adherence to the Mediterranean diet among patients with metabolic syndrome. CBT is a group of short-term psychotherapy methods that aims to help patients to understand relationships between their thoughts, feelings, and behaviors [52, 53]. It encourages patients to change maladaptive cognitions and behaviors [54]. Through cognitive and behavior therapies, intervention group patients learned to identify and challenge automatic thoughts, relieve negative emotions, and alter underlying thoughts. Consequently, their dysfunctional thoughts were replaced with functional thoughts, enhancing health-related behaviors [53]. Therefore, CBT-based intervention is an approach that may promote behavioral change in T2DM patients with comorbid MS.

### Effects of CBT-based intervention on quality of sleep

There were significant improvements in the subjective sleep quality and daytime dysfunction subscales of PSQI in the intervention group. Similarly, Zuo and colleagues [55] also demonstrated that patients in the CBT intervention group had lower PSQI scores (better sleep quality) than those in the control group. The positive result might be related to the CBT techniques used to improve sleep quality in this study, including sleep health education, sleep restriction, stimulus control, and cognitive restructuring [56]. The patient sleep–wake biological rhythm was gradually established, resulting in an improved quality of sleep. Our findings further demonstrated that CBT-based intervention may also be a beneficial therapy for sleep disturbances in T2DM patients with comorbid MS as well as in the general population [56].

### Effects of CBT-based intervention on physiological variables

This study argued that the intervention group decreased more in BMI, HbA_1c_, SBP, and DBP, but not to a significant level when compared with the control group at the post-intervention time. Likewise, Jenkinson and colleagues also demonstrated that there was not a significant effect of CBT on HbA_1c_. In addition, one previous study [57] indicated that psychological interventions were not beneficial in reducing the BMI and blood pressure in type 2 diabetes patients, but were beneficial in improving dietary behaviors. One potential explanation could be that since CBT is a type of psychotherapy, its impact on relieving symptoms of depression and anxiety, as well as improving health-related behaviors, may be more significant than its effect on physiological variables.

### Strengths, limitations, and implications

An increasing number of studies have applied CBT in chronic disease intervention research with satisfying results, including hypertension [58], diabetes [59], cancer [60], and chronic pain [46]. However, this was the first study to investigate the effectiveness of CBT in patients with comorbidities and the results were promising. Findings showed that CBT-based intervention was beneficial in improving depression symptoms and diabetes self-care behaviors in T2DM with comorbid MS. Additionally, intervention group patients had a larger downward trend in physiological variables. This study further demonstrates that CBT was a beneficial approach in patients with comorbidities and has implications in theory for developing further intervention programs on diabetes.

However, there are some limitations in the present study. Firstly, this study lacked a follow-up period, so it was unable to assess the long-term effects of CBT, but the short-term effects of CBT were also promising and shed light on future studies on diabetes. Secondly, this study adopted a combination of face-to-face and online intervention modes. The adherence of patients to online intervention sessions was an unpredictable factor, which may have confounded results. However, several approaches were taken to ensure patient compliance, including the after-session quiz and feedback, group discussions, and telephone follow-up during the intervention time. Furthermore, the therapist and patients were aware of the grouping results in advance, and the subjective factors of knowing these may have biased the results.

In general, CBT has been proven to be a beneficial method in relieving negative emotions and promoting behavior change in chronic disease management. However, there is still no standardized CBT intervention manual for chronic diseases, including the intervention time, the number of sessions, the proper time for one session, or the appropriate backgrounds of therapists. Therefore, future research should be focused on developing a standardized manual for diabetes, so that general practitioners, nurses, and other medical service staff could benefit from the manual. Moreover, the quality of interventions using CBT will also be improved.

## Conclusions

This study demonstrated that CBT-based interventions were beneficial in relieving patient depression symptoms. Moreover, the overall self-care behavior of patients improved more in the intervention group. In addition, CBT-based intervention was more helpful in enhancing the sleep quality of patients. Finally, patients in the intervention group decreased more in BMI, HbA_1c_, SBP, and DBP, but not to a significant level. It can be concluded that CBT-based intervention is conducive to relieving negative emotions and promoting behavior change in T2DM patients with comorbid MS.

## Supplementary Information

**Additional file 1: Figure S1.** Intervention flow chart. **Table S1. **Patient participation details. **Table S2. **Intervention session contents.

## Data Availability

The datasets used and/or analyzed during the current study are available from the corresponding author upon reasonable request.

## Abbreviations

CBT: Cognitive behavior therapy
DM: Diabetes
T2DM: Type 2 diabetes
MS: Metabolic syndrome
BMI: Body mass index
SBP: Systolic blood pressure
DBP: Diastolic blood pressure
HbA_1c_: Glycated hemoglobin
FBG: Fasting blood glucose
TG: Total triglycerides
TC: Total cholesterol
HDL–C: High-density lipoprotein
LDL–C: Low-density lipoprotein
PHQ-9: Patient Health Questionnaire-9
GAD-7: General Anxiety Disorder 7-Item
SDSCA: The Summary of Diabetes Self-Care Activities
PSQI: The Pittsburgh Sleep Quality Index
