# Supplementary material for: The evaluation of cognitive-behavioral therapy-based intervention on type 2 diabetes patients with comorbid metabolic syndrome: a randomized controlled trial
Source: Diabetol Metab Syndr. 2023 Jul 17;15:158. doi: 10.1186/s13098-023-01100-2 (PMC10351126; doi:10.1186/s13098-023-01100-2)
Supplement: Supplementary file 1 — Additional file 1: Figure S1. Intervention flow chart. Table S1. Patient participation details. Table S2. Intervention session contents. [file 13098_2023_1100_MOESM1_ESM.docx]

**
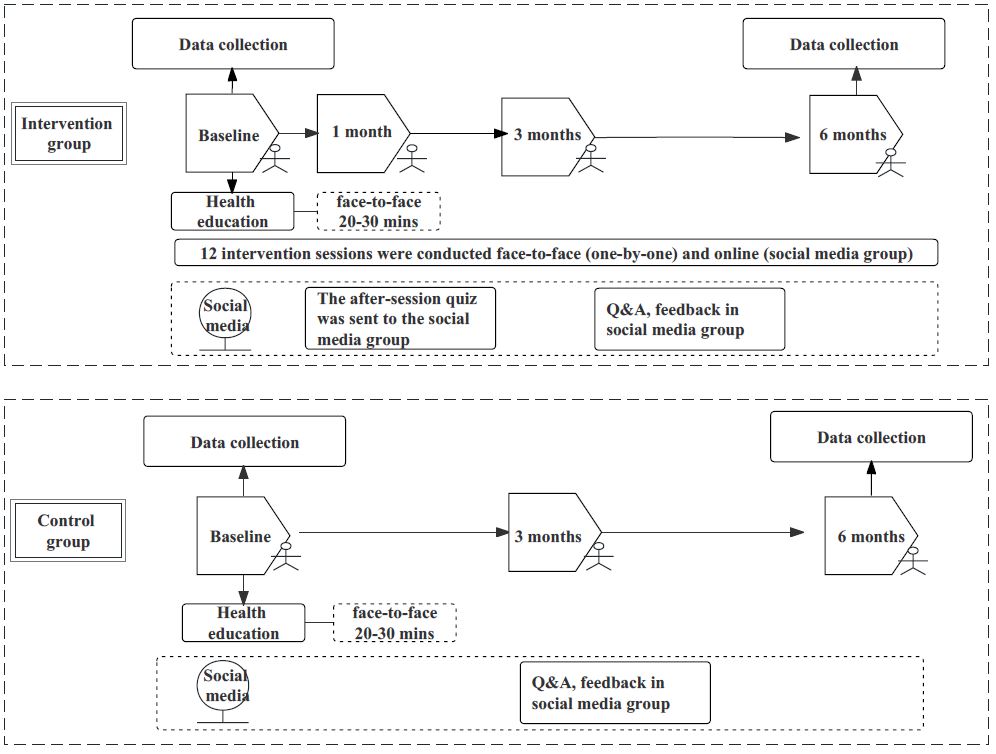
**

**Supplementary Figure 1 |** Intervention flow chart.

**Supplementary Table 1** Patient participation details.

| Intervention group | Total number | Drop-out number | Session1 | Session2 | Session3 | Session4 | Session5 | Session6 | Session7 | Session8 | Session9 | Session10 | Session11 | Session12 |
| --- | --- | --- | --- | --- | --- | --- | --- | --- | --- | --- | --- | --- | --- | --- |
| Group1 | 11 | 0 | 11 | 6 | 6 | 3 | 7 | 7 | 7 | 10 | 4 | 8 | 8 | 11 |
| Group2 | 9 | 1 | 9 | 7 | 7 | 2 | 8 | 8 | 8 | 8 | 3 | 6 | 6 | 8 |
| Group3 | 12 | 2 | 12 | 9 | 9 | 3 | 8 | 9 | 9 | 6 | 4 | 8 | 8 | 10 |
| Group4 | 17 | 2 | 17 | 12 | 12 | 4 | 14 | 14 | 14 | 12 | 5 | 12 | 12 | 15 |
| Group5 | 14 | 2 | 14 | 12 | 12 | 3 | 11 | 12 | 12 | 12 | 4 | 12 | 12 | 12 |
| Group6 | 17 | 4 | 17 | 9 | 9 | 4 | 9 | 9 | 9 | 9 | 5 | 12 | 12 | 13 |
| Group7 | 13 | 0 | 13 | 11 | 11 | 3 | 12 | 12 | 13 | 13 | 4 | 13 | 13 | 13 |
| Group8 | 17 | 3 | 17 | 12 | 12 | 4 | 12 | 14 | 14 | 13 | 5 | 13 | 13 | 14 |
| Group9 | 20 | 4 | 20 | 15 | 15 | 4 | 14 | 15 | 15 | 15 | 6 | 15 | 15 | 16 |
| Group10 | 18 | 3 | 18 | 14 | 14 | 4 | 13 | 13 | 13 | 14 | 5 | 14 | 14 | 15 |

**Supplementary Table 2 |** Intervention session contents.

| Session | Aim |
| --- | --- |
| 1. Develop a treatment alliance and set treatment goals together | Build treatment alliances, introduce CBT therapy |
| 2. Psychological education and normalization—develop a list of problems related to DM/MS | Build treatment alliances, learn about DM and MS, and make a list of the problems caused by the disease |
| 3. Use exposure therapy—common complications of DM | Understand the causes, primary symptoms, and preventive measures for common complications of DM |
| 4. Use Socrates Questions—strengthen self-monitoring of blood glucose | Understand the significance of self-monitoring of blood glucose, master the methods of self-monitoring of glucose, and develop a self-monitoring of blood glucose habit |
| 5. Negative thinking substitution—maintain dietary nutrition and common diet-related distortions | Understand the relationship between diet and health, master the principles of a healthy diet for diabetes, develop the diet plan, and understand dietary-related cognitive distortions |
| 6. Negative thinking substitution—maintain moderate exercise and common exercise-related distortions | Understand the relationship between exercise and health, choose the suitable exercise intensity and pay attention to safety when doing exercise, understand common exercise-related distortions |
| 7. Negative thinking substitution—adherence to the medication and common medication-related cognitive distortions | Comprehend the commonly used medications for diabetes, including their types, primary mechanisms of action, side effects, and necessary precautions. Acquire knowledge regarding the distinctive features of various insulin types and their storage methods. Gain proficiency in the proper techniques of insulin injection. Recognize prevalent misconceptions regarding drug treatment for diabetes |
| 8. Use the cognitive triangle—identification and diagnosis of automatic thinking related to DM/MS | Understand automatic thinking, identify common thinking traps for DM/MS, and learn about the "cognitive triangle" |
| 9. Use cognitive technology—identification, diagnosis, and replacement of distorted cognitive perceptions associated with DM/MS | Deepen understanding of the "cognitive triangle," identify common distortions of cognition in DM/MS, and attempt to substitute the distorted cognition |
| 10. Use behavioral technology—identification, diagnosis, and replacement of distorted cognition associated with DM/MS | Review the "cognitive triangle" to enhance the understanding of the interrelations between thoughts, emotions, and behaviors, understand how to relieve negative emotions from the perspective of behavior, master some relaxation methods |
| 11. Use exposure therapy—stop smoking and limit alcohol and develop good habits | Recognize the health damage of tobacco and excessive drinking, recognize the importance of good sleep, and develop good habits |
| 12. Psychological education and normalization—prevention of diabetes | Understand the ways to prevent DM, review the core contents of this intervention, and get feedback |
